# Exquisite design of injectable Hydrogels in Cartilage Repair

**DOI:** 10.7150/thno.46450

**Published:** 2020-08-02

**Authors:** Jiawei Wu, Qi Chen, Chao Deng, Baoping Xu, Zeiyan Zhang, Yang Yang, Tingli Lu

**Affiliations:** 1 Key Laboratory for Space Bioscience and Biotechnology, Northwestern Polytechnical University School of Life Sciences.; 2 Key Laboratory of Resource Biology and Biotechnology in Western China, Ministry of Education. Faculty of Life Sciences, Northwest University, 229 Taibai North Road, Xi'an 710069, China.; 3 Department of Cardiovascular Surgery, The First Affiliated Hospital of Xi'an Jiaotong University, 277 Yanta West Road, Xi'an 710061, Shaanxi, China.

**Keywords:** injectable hydrogels, cartilage repair, polymers, gelation

## Abstract

Cartilage damage is still a threat to human beings, yet there is currently no treatment available to fully restore the function of cartilage. Recently, due to their unique structures and properties, injectable hydrogels have been widely studied and have exhibited high potential for applications in therapeutic areas, especially in cartilage repair. In this review, we briefly introduce the properties of cartilage, some articular cartilage injuries, and now available treatment strategies. Afterwards, we propose the functional and fundamental requirements of injectable hydrogels in cartilage tissue engineering, as well as the main advantages of injectable hydrogels as a therapy for cartilage damage, including strong plasticity and excellent biocompatibility. Moreover, we comprehensively summarize the polymers, cells, and bioactive molecules regularly used in the fabrication of injectable hydrogels, with two kinds of gelation, i.e., physical and chemical crosslinking, which ensure the excellent design of injectable hydrogels for cartilage repair. We also include novel hybrid injectable hydrogels combined with nanoparticles. Finally, we conclude with the advances of this clinical application and the challenges of injectable hydrogels used in cartilage repair.

## Introduction

Cartilage damages are responsible for progressive joint pain and disability in millions of people worldwide and occur as a result of trauma, congenital anomalies, and skeletal diseases 1, 2. Clinical studies have shown that 60% of patients examined by knee arthroscopy exhibit cartilage damage and 15% of people over 60 years old present clinical symptoms of such damage 3, 4. Although cartilage is considered to be a simple tissue, there are great limitations in the current treatments for its damage. First, cartilage's high matrix-to-cell ratio, avascular nature and consequent lack of access to a pool of potential reparative cells and humoral factors create an environment that is challenging to heal 1, 5, 6. Moreover, cartilage defects and injuries can cause posttraumatic osteoarthritis (OA), which increases the difficulty of healing cartilage damage.

Cartilage restoration surgical procedures have been the common treatment of chondral lesions in the young adult population. However, these surgeries just provide temporary symptomatic relief and not a cure, without regenerating functional cartilage. Advances in tissue engineering have made it possible to develop biological substitutes that can restore, maintain or improve tissue function for therapeutic purposes. Since the 1990s, a variety of biomaterials have been investigated and tested for cartilage tissue engineering applications 7-13. Among all the biomaterials, injectable hydrogels have gained widespread attention, particularly for their use as scaffolds in cartilage and bone tissue engineering. Due to their remarkable characteristics, including flexibility and versatility in fabrication, variety in composition, high moldability in shape, excellent biocompatibility and similarity to extracellular matrix (ECM), injectable hydrogels are believed to become promising materials in the biomedical fields 14-16.

The following review aims to illuminate the application of injectable hydrogels in cartilage repair. First, we introduce the basics of cartilage, focusing on articular cartilage. We then propose the functional and fundamental requirements for injectable hydrogels applied in cartilage repair. Then, we summarize the advantages of injectable hydrogels in tissue engineering. Moreover, we focus on the design of proper injectable hydrogels for cartilage repair, including appropriate materials (polymers, cells, and bioactive molecules) and fabrication techniques (chemical and physical crosslinking). Afterwards, we review a novel hybrid hydrogel that is combined with nanoparticles and has become attractive for the further development of injectable hydrogels. In addition, we also explore the advances of injectable hydrogels in specific clinical applications of cartilage repair. Finally, we discuss the challenges of injectable hydrogels during application. Perspectives on future injectable hydrogels for cartilage tissue engineering are also discussed. Collectively, this review may be useful for developing injectable hydrogels for future therapies in cartilage damage.

## Cartilage and articular cartilage

### Composition and classification of cartilage

Cartilage is classified as a special connective tissue that contains no blood vessels or lymphatic vessels. Thus, its nutrition relies on diffusion from the surrounding tissues. Furthermore, cartilage comprises chondrocytes and ECM. Based on the difference of ECM, it is possible to divide cartilaginous tissues into three types: hyaline, fibro- and elastic cartilage. The cartilage, the matrix of which is rich in glycosaminglycans (GAGs) and highly adherent collagen fibers (mainly type II collagen), is considered to be hyaline cartilage. The matrix in fibrocartilage comprises densely braided collagen fibers, which make fibrocartilage highly resistant to compression. In contrast to hyaline cartilage, fibrocartilage contains higher levels of type I collagen than type II collagen and only a small proportion of GAGs. Moreover, the fibrillar component distributes prevalently in the elastic cartilage ECM, in which type II collagen and elastic fibers are densely branched in multiple directions.

Notably, fibrocartilage is mainly distributed in the intervertebral disc, articular disc and pubic symphysis, as well as elastic cartilage in the auricle and epiglottis. Hyaline cartilage exists widely. Adult articular cartilage, costal cartilage and some cartilage of the respiratory tract all belong to hyaline cartilage, making it important in clinical research. Thus, here, we will focus primarily on hyaline cartilage, as it is important in clinical research and has been formed using the current reparative and restorative clinical procedures.

### Classification and zoning of articular cartilage

Articular cartilage is considered to be the typical hyaline cartilage and comprises chondrocytes and ECM. Chondrocytes are differentiated from mesenchymal cells, which can form and maintain the ECM in cartilage. The matrix in cartilage is in a gel state, consisting of type II collagen, proteoglycan and water. Type II collagen accounts for 90%-95% of collagen in articular cartilage, which guarantees the shear resistance of the articular cartilage. In addition, proteoglycan and hyaluronic acid cross-link to form a brush-like structure that contains a large amount of water, resulting in a better elasticity and compressive resistance of articular cartilage.

**Figure 1:**
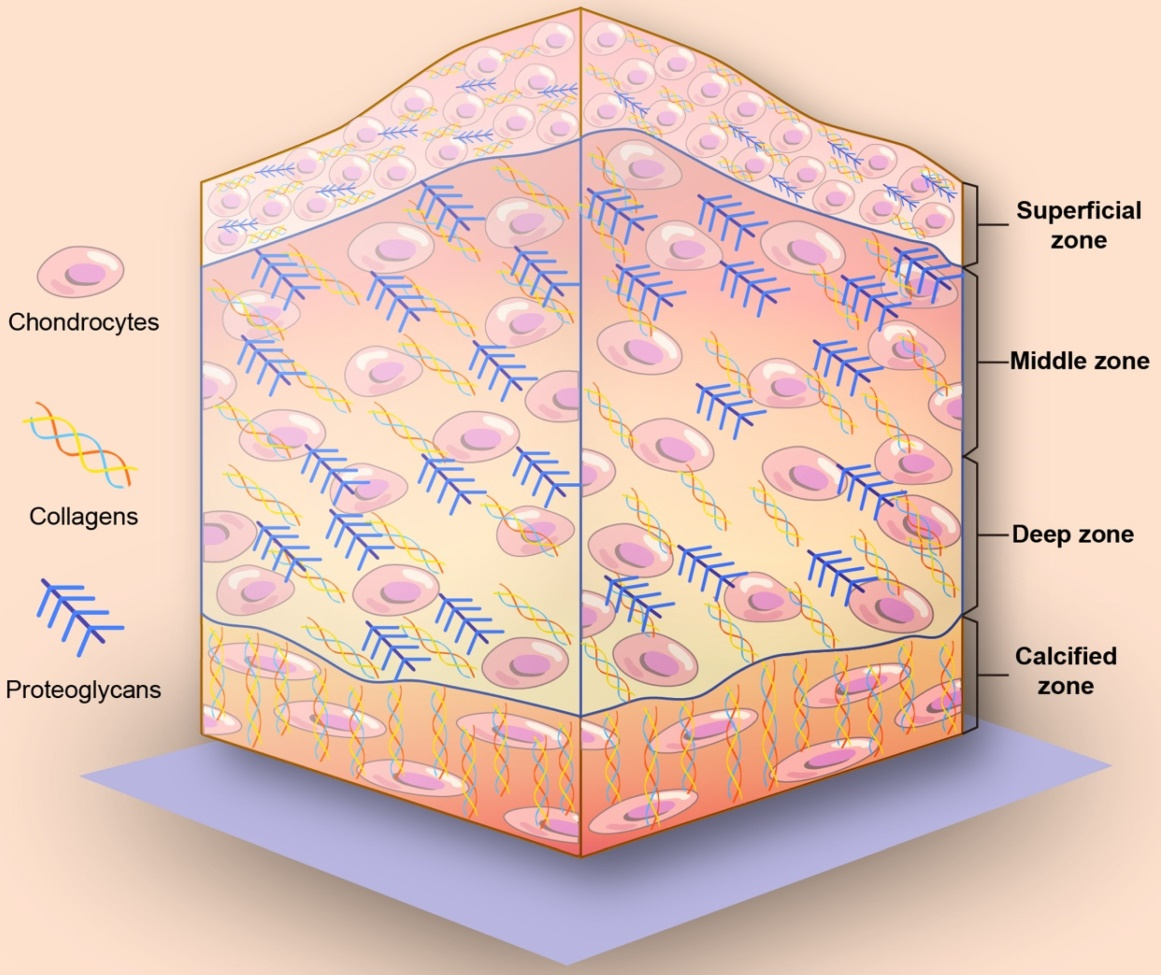
** Schematic illustration of the depth-dependent architecture of articular cartilage tissue.** From the superficial zone to the deep zone, the proteoglycan content gradually increases. In the superficial cartilage, the collagen and chondrocytes are arranged in parallel with the joint surface. In the middle zone, collagen fibers are unaligned and tangential to the cartilage surface. In the deep zone, collagen fibers are arranged radially. In the calcified zone, collagen fibers tend to arborize with little organization and mineralization.

On the basis of collagen fiber alignment and proteoglycan composition, articular cartilage can be divided into four different zones: a superficial zone, a middle zone, a deep zone, and a calcified zone (**Figure 1**) 13, 17, 18. From the superficial zone to the deep zone, the proteoglycan content gradually increases. The collagen and chondrocytes in the superficial cartilage are arranged in parallel with the joint surface. In the middle zone, collagen fibers are unaligned and tangential to the cartilage surface, while collagen fibers are arranged radially in the deep zone. Finally, close to the bone is the calcified zone, and in the calcified zone, collagen fibers tend to arborize with little organization and mineralization. The mechanical properties are summarized in **Table 1.**

There is no clear boundary between the layers of articular cartilage, and the changes are transitional. Furthermore, the hierarchical changes of articular cartilage structure are suitable for the mechanical environment of articular cartilage. The shallow layer is mainly adapted to shear load, and the deeper the structure is, the more suitable it is to bear the compression load. The function of the calcified layer and longitudinal fiber plays a role in firmly fixing the articular cartilage surface to the subchondral bone 19.

### Damage and repair of articular cartilage

Differences in the type of injury and the repair response distinguish three classes of articular surface injuries: 1) damage to the joint surface that leaves the articular surface intact but causes internal chondral damage and may cause subchondral bone injury, 2) mechanical disruption of the articular surface limited to articular cartilage, and 3) mechanical disruption of articular cartilage and subchondral bone 12.

The pathological changes in the internal chondral damage are the cartilage matrix fracture, but the survival chondrocytes can enhance their synthesis function to repair the tissue. Defects limited to articular cartilage cannot be self-healing, as the stem cells in the bone marrow cannot be extracted. As a result of mesenchymal stem cells in the bone marrow migrating to the cartilage damage and producing repaired fibrocartilage tissue, mechanical disruptions of articular cartilage and subchondral bone can be repaired to some extent 20. Although the repaired tissue is far inferior to healthy cartilage in terms of biomechanical properties and durability, it can cover and protect the subchondral bone in the defect area and reduce the formation of cartilage wear and free debris.

According to the patient's cartilage injury, a variety of different treatment methods can be used clinically. In general, the treatment of articular cartilage mainly focuses on stimulating surgery (stimulating the self-repair of articular cartilage) and transplanting surgery (using autologous or allogeneic cell or tissue) 21. The former includes joint cleaning, joint debridement, cartilage grinding and forming, drilling, microfracture, osteotomy, and joint traction. The latter includes autologous or allogeneic cartilage transplantation, periosteum transplantation and perichondrium transplantation, autologous chondrocyte implantation (ACI), osteochondral transplantation and other transplantation repair techniques.

Joint cleaning and debridement are commonly used to reduce pain in patients with joint disease, but the mechanism is still unknown 22. Moreover, there is no obvious physiological or pathological evidence to show that the two are beneficial for cartilage repair 23. Drilling, grinding, and microfracture mainly occur through the subchondral bone damage during the surgical operation, stimulating the production of coagulation clot to induce fibrous tissue block to promote the regeneration of the articular cartilage. The effects of these methods are related to patients' age, degree of joint damage, patients' activities and other factors, so the repairs of these surgical methods have different reports 24.

Periosteum and perichondrium transplantation is a common clinical method for articular cartilage repair. It has been reported that there is no significant difference in the repair effect of periosteum and perichondrium, but the periosteum has more obvious clinical application advantages, including extensive source and easy regeneration 25, 26. However, due to the existence of adhesion and fixation difficulties, the application of this method is limited. Similarly, potential calcifications will also lead to the reduction of the repair effect. Thus, it is difficult to obtain a long-term stable repair effect using this method 27-29.

ACI is a two-stage, typically second-line intervention after at least arthroscopic debridement or microfracture has produced an inadequate clinical outcome 30. In ACI, cultured autologous chondrocytes are used to resurface symptomatic chondral defects with hyaline or hyaline-like cartilage 31. Although the method has yielded some successful results in clinical applications, some molecular mechanism studies have shown that the expression levels of type II collagen and several important transcription factors related to the proliferation and differentiation with ACI are low 32-34.

Notably, tissue engineering, which has begun appearing in the public and is gaining increasing attention, forms tissues by the combinations of cells, scaffolds, and matrices *in vitro* for implantation. The avenue of tissue engineering explores the use of cells, scaffolds, and biological factors alone or in combination toward the repair, restoration and replacement of tissues 5.

## Injectable hydrogels in cartilage repair

Recently, hydrogels, especially injectable hydrogels, acting as three-dimensional scaffolds have received increasing attention for articular cartilage tissue engineering, and they may become a promising treatment for the above three classes of articular injuries.

### Principal function and fundamental requirements of hydrogels for cartilage repair

**Figure 2:**
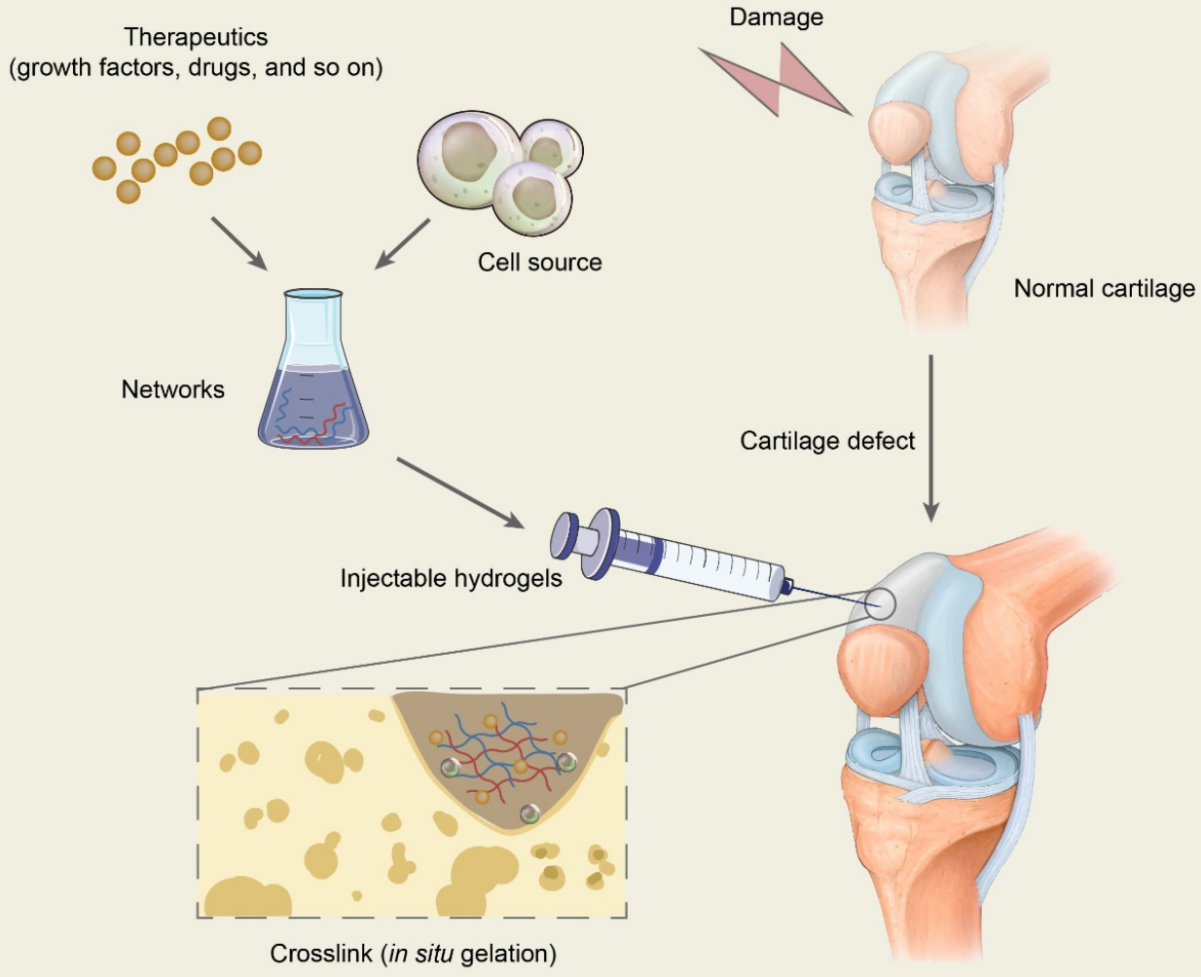
** Schematic diagram of the application of injectable hydrogels for cartilage repair.** Therapeutics including drugs and bioactive molecules are usually encapsulated in the networks, which are formed by polymer-based injectables. All the ingredients constitute the precursor liquid solution and are injected into target sites of cartilage defects. The injectable hydrogels will gel* in situ* through chemical reactions or by physical factor induction and are expected to repair the cartilage defects.

The scaffold in tissue engineering is designed to create a three-dimensional (3D) microenvironment that resembles specific tissues, stimulating natural tissue regeneration by promoting cell-to-matrix interactions and cell-to-cell interactions, leading to cell differentiation and tissue growth 35. Hydrogels are 3D network structures that are formed by hydrophilic homopolymers or copolymers crosslinked either through covalent bonds or held together via physical intramolecular and intermolecular attractions. Cells and bioactive molecules or drugs are usually encapsulated in the networks formed by polymers and are mixed with injectable hydrogels to constitute the precursor liquid solution being injected into target sites, which will gel* in situ* and is expected to repair the cartilage defects (**Figure 2**). In conclusion, hydrogels in cartilage repair not only are a support for cell growth but also promote tissue formation by transporting nutrients, similarly providing appropriate mechanical strength 35, 36.

Hydrogels, including injectable hydrogels, belong to scaffolds in the tissue engineering for cartilage repair. Thus, some fundamental requirements of scaffolds need to be taken into account for future applications, including biocompatibility (no toxicity, no inflammation and immunity), biodegradability (appropriate degradation time in the case of biodegradable materials), mechanical properties (support for new tissues), and plasticity (to make the desired shape) 37.

### Advantages of injectable hydrogels

Injectable hydrogels are a new type of hydrogel system with certain fluidity that can be implanted into the body by injection. Moreover, injectable hydrogels have the ability to set* in situ* by physical or chemical crosslinking to form 3D scaffolds. Injectable hydrogels have been recognized as a promising material in cartilage tissue engineering for several advantages over other cartilage restoration techniques, including simple operation, strong plasticity, good biocompatibility, and excellent biodegradability, among others.

#### Compared with conventional treatment strategies for cartilage damages

As previously indicated, the present treatments exhibit some efficacy for cartilage repair, but there are still certain limitations, including poor integration with healthy cartilage, lack of nutrients, and high technical requirements for surgery. Therefore, injectable hydrogels applied into tissue engineering exhibit some advantages over conventional strategies.

##### Minimally invasive surgery

Injectable hydrogels are increasingly used in the field of minimally invasive surgeries (MIS), which are practiced in most surgical fields, including orthopedic, neurological and cardiovascular procedures. These procedures are performed through small incisions and are associated with a lower risk of postoperative infection and reduced postoperative pain 38. In addition, surrounding tissue damage and postoperative pain and scar size can be minimized to a large extent, with reduced medical costs and shorter hospital stays 39.

##### Simple operation

Injectable hydrogels can be injected into the body as a prepolymer solution and then polymerize* in vivo*, which simplifies implantation during surgeries 40. Moreover, the simple injection significantly decreases patients' compliance relative to preformed hydrogels that must be crosslinked *in vitro* and then surgically implanted *in vivo*. As the implants are formed* in situ* at the defect site when processing in the operating room, the surgical time can be shortened.

##### Strong plasticity

Injectable hydrogels can be cast into practically any shape, size, or form, which gives them the ability to appropriately fill irregularly shaped defects 39 making up for the defects of traditional strategies that sometimes cannot form ideal-shaped cartilage in the damage area.

#### Compared with other scaffolds in tissue engineering

In terms of the morphological properties, cartilage tissue engineering scaffolds can be divided into two types: solid scaffolds and hydrogels 41. Traditional solid scaffolds, which are mainly made of metal, calcium phosphate ceramics, or glass, have the properties of bone conduction but not bone induction. One of the limitations of metal scaffolds is that they can release toxic metallic ions through corrosion or wear, which can lead to an inflammatory cascade and allergic reactions 42-44. Another drawback is a lack of biometrics on the material's surface, making it hard to metabolize 44. In addition, the metal materials have stress shielding in bone tissue, reducing the stress level of bone and increasing the risk of postoperative fracture 44. For inorganic materials, including glass and ceramic scaffolds, the disadvantages of intrinsic brittleness, low resistance to crack propagation, and low bending strength limit their development 45. Furthermore, the nonbiodegradability of the traditional scaffolds in the natural environment is also a challenging defect 46. Solid porous scaffold materials include honeycomb, porous entity, mesh, sponge, nonwoven texture materials, etc. 47. Their advantages include that they are easy to shape, have good strength, and the degradation rate can be adjusted by the composition and molecular weight. However, studies have shown that most cells in porous scaffolds adhere to and extend only on the surface of the pore wall in the scaffolds, thus forming a single layer of cells, which is different from the morphology, number and distribution of cells in natural cartilage 48, 49. Hydrogels have made up for these defects due to their highly aqueous three-dimensional network structure. In addition to their special structure, injectable hydrogels also have other advantages over solid porous scaffold materials.

##### Highly porous three-dimensional structures

Injectable hydrogels are 3D architectures with high porosity, which makes it possible to encapsulate a large number of cells in the materials and provides a good site for cell adhesion, proliferation and specific differentiation. One of the unique advantages of hydrogels is that they encapsulate the cells, allowing them to live in a three-dimensional environment that allows the chondrocytes to maintain their phenotype.

##### Similarity to ECM

Injectable hydrogels are able to absorb huge amounts of water or biological fluids and swell readily without dissolving. Hydrogels have high hydrophilicity, owing to the presence of hydrophilic moieties such as carboxyl groups, amides, amino groups and hydroxyl groups distributed along the backbone of the polymeric chain 50. The normal articular cartilage is a liquid-solid material with biphasic properties. The chondrocytes in the cartilage tissue are spherical and surrounded by a matrix comprising collagen, proteoglycan, hyaluronic acid (HA), and other components. In the swollen state, the hydrogel is soft and rubbery, similar to living tissues to a great extent, providing an almost normal chondrocyte growth environment 35, 41. Proper mechanical properties maintain the function and vitality of the cells, supporting the new tissue until it is formed.

##### Incorporation of biological signals

For injectable hydrogels, gelation occurs under very mild physiological conditions, allowing successful encapsulation of cells and biologically active proteins or peptides for regenerative applications 51. Moreover, the hydrogels can bind to bioactive cues such as growth factors to provide chemical signals to the cells, which can stimulate cell proliferation.

##### *In situ* gelation

Some solid porous scaffold materials such as sponges have difficulties in implantation due to their shape, the need for extensive substrate preparation, and the necessity of removal of a relatively large amount of healthy tissue 37. Such polymeric hydrogels formed on the rationale of phase transition from a solid state at ambient conditions to a gel phase on exposure to physiological conditions are collectively termed as injectable or* in situ*-forming hydrogels 52. These hydrogels are superior to preformed scaffolds in terms of improved patient compliance and overcome the risk of implant migration. Other advantages include simple cell encapsulation and ease of clinical implementation via a minimally invasive route for the treatment of geometrically irregular, larger and deeper lesions 53-55. However, injectable hydrogels are injected into the body as a liquid followed by gelation *in situ* through the control of factors such as pH and temperature. They have not only overcome the implantation difficulties of solid porous scaffolds but also enabled the spatiotemporal distribution of functional bioactive factors and viable cells 52, 56, 57.

##### Excellent biocompatibility

The term “biocompatibility” has been defined as “the ability of a material to locally trigger and guide nonfibrotic wound healing, reconstruction and tissue integration” 58. Injectable hydrogels are nontoxic and fail to cause immune and inflammatory responses, which enables their persistence and efficacy *in vivo*. Furthermore, injectable hydrogels are similar to the native ECM in physical properties, thus providing a beneficial natural microenvironment for cell migration, proliferation and adhesion 35. With good biocompatibility, injectable hydrogels may be a suitable platform to promote the regeneration of cartilage tissue.

##### Good biodegradability

The biodegradability of hydrogels is critical for biomedical applications, as biomedical applications require controlled absorption or local dissolution *in vivo* to facilitate cellular activity and promote tissue repair. Most hydrogels are able to undergo local or bulk dissolution by hydrolysis, proteolysis or disentanglement 16.

## Design of injectable hydrogels

The design of a suitable injectable hydrogel that incorporates excellent biocompatible, biodegradable, and mechanical properties for clinical repair of damaged cartilage is a challenge of significant medical interest, which remains to be achieved with an adequate gelation time 39. These factors are why the material used for preparation and the fabrication method chosen are quite important.

### Polymers

Injectable hydrogels applied in tissue engineering must meet a range of design criteria, including biodegradation, porosity, biocompatibility, and cell adhesion 50. Moreover, these standards are closely related to the polymers used to fabricate the injectable hydrogels. Gradually, injectable hydrogels are divided into natural or synthetic cross-linked polymers, or a combination of both.

#### Natural polymers

Natural polymers can be roughly classified into three categories: polymers based on polysaccharide, protein, and proteonucleotide. Natural materials are appealing for the preparation of injectable hydrogels due to their similarity to the natural cartilage ECMs, thus obtaining excellent biocompatibility and biodegradability 14, 15, 59. Furthermore, they are favored by their affinity toward other biomolecules and their non-immunogenicity 60, 61.

##### Polysaccharide-based polymers

*HA:* As a high-molecular-weight linear polysaccharide, HA comprises disaccharide units of glucuronic acid and N-acetylglucosamine. It can interact with cells, notably with chondrocytes, through surface receptors such as CD44 and RHAMM, which play important roles in cartilage formation, mesenchymal cell migration, condensation, chondrogenic differentiation, and cartilage homeostasis maintenance 62-64. In addition, HA is found in soft tissue and synovial fluid, such as cartilage ECM. Due to its natural presence in mammalian tissue and its nontoxicity, HA has been approved by the U.S. Food and Drug Administration (FDA) for implantation in humans, mostly as a space filler (Restylane®, Juvederm®) and for viscosupplementation (Monovisc^TM^, Hymovis®) 38. Furthermore, HA provides lubrication and resistance to articulating surfaces, promotes wound healing and tissue regeneration, and increases osteoblastic bone formation. Consequently, it is a potential material for cartilage tissue repair 18, 65, 66.

***Chitosan:***Chitosan is a linear polysaccharide comprising of d-glucosamine and N-acetylglucosamine units, is derived from the deacetylation of natural chitin and is found in the shells of shrimps and crabs. It has been described to exhibit great properties, including its biocompatibility, biodegradability, hemostatic activity, and antibacterial activity 67. Particularly, it is structurally similar to naturally occurring GAGs 92, making chitosan-based hydrogels great candidates as injectable materials for cartilage repair 68-71. Moreover, chitosan has been FDA-approved mostly for hemorrhage control (hemostasis bandages) (HemCon®) 38. Recently, chitosan-based materials have been used to accelerate wound healing and to support the proliferation and differentiation of osteoblasts and chondrocytes.

***Alginate:***Alginate is a natural polysaccharide extracted from brown algae (Phaeophyceae) consisting of guluronic and maluronic acids 72-75. Alginate displays interesting properties, including favorable scaffold formation, biocompatibility, non-immunogenicity, and nontoxicity, which ensures alginate a common application in cartilage tissue engineering 73-76. Moreover, alginate is FDA-approved 77, and some alginate hydrogels are already commercialized, mostly as 3D cell culture matrices (AlgiMatrix^TM^, QuickGel^TM^) 78 or for wound dressing applications (Nu-Gel^TM^) 38.

***Cellulose:***Cellulose is a neutral polysaccharide comprising a linear chain of β-1,4-D-glucose units. Currently, cellulose and its water-soluble derivatives have been applied in the fabrication of hydrogels, which includes methyl cellulose (MC), carboxymethyl cellulose (CMC), hydroxypropyl cellulose (HPC) and hydroxypropylmethyl cellulose (HPMC) 38. Although cellulose-based hydrogels are not degraded easily *in vivo* without cellulases, their biocompatibility, water-soluble property, easily sterilized property, and nontoxicity make them great candidates as injectable materials for cartilage repair.

##### Protein-based polymers

***Collagen:***Collagen is the most abundant protein found in cartilage, bone, skin, ligaments, and connective tissue of the body, accounting for one-third of the total protein amount 79, 80. There are at least 19 types of collagen, including type I, type II, type III, and type V 39, 81. Like other natural polymers, collagen has its inherent advantages, such as excellent biocompatibility and biodegradability. Additionally, the low antigenic property and the ability of inducing chondrogenic differentiation have attracted increasing interest in tissue engineering applications, particularly in the field of cartilage repair 82-85.

***Gelatin:***Gelatin is a fraction of polypeptides derived from partial hydrolytic degradation of collagen, containing arginine-glycine-aspartic acid sequences that can directly interact with integrins on cell surfaces and promote cell adhesion 15. Gelatin is inexpensive and has superior water solubility, processability and lower immunogenicity compared to collagen, as well as inherent bioactivity, which makes it an attractive precursor for the fabrication of hydrogels 86. Additionally, its cartilage reparation properties suggest it will become a promising material to prepare injectable hydrogels applied in cartilage repair.

***Elastin:***Elastin is an insoluble, highly cross-linked, polymeric, and elastic protein that can be divided into two types: elastin I is present in ligaments, aorta and skin, while elastin II is present in cartilage. Elastin molecules can be arbitrarily crimped, and the molecules are crosslinked into a network by covalent bonds. Recently, elastin-based biomaterials have been widely used in tissue engineering, especially in developing injectable hydrogels for cartilage tissue engineering, as their improved local elasticity can facilitate cellular interactions and signaling 87, 88.

***Fibrin:***Fibrin is a natural fibrous protein involved in blood clotting that can enhance cell attachment, mobility, proliferation and differentiation. However, fibrin presents low mechanical stiffness and fast degradation and is commonly used in combination with other materials. Notably, as alginate microbeads exhibit excellent stability and biocompatible properties, they are often combined with fibrin to fabricate injectable hydrogel systems for tissue regeneration 89.

##### Proteonucleotide-based polymers

***Deoxyribonucleic acid (DNA):***DNA is a long-chain polymer comprising repeating arrangements of nucleotides. DNA can be physically gelatinized by hydrogen bonds, van der Waals forces, and the winding of DNA molecular chains. Moreover, some enzymes such as DNA ligase help DNA realize chemical gelatinization 90, 91. In addition, DNA is characterized by good biocompatibility, biodegradability, molecular recognition, nanometer size control, and specific coding 92. In the past 30 years, DNA has received considerable attention as a promising material, including as a genetic material for biological systems and as a biomaterial for the construction of nanostructures, because of its precise base-paring recognition, designable sequence, and predictable secondary structure 90, 91, 93, 94. DNA hydrogels made of well-defined small building blocks are a type of tenuous, semiflexible polymeric network that consists of precisely designed synthetic nucleotide strands as chemical or physical cross-linkers 95-97. Furthermore, DNA-based hydrogels have been proved to maintain the original biological characteristics of DNA and have the characteristics of ordinary gels, such as shape plasticity, certain mechanical strength, transport materials, and other characteristics 16.

Though natural polymers have been widely investigated, their mechanical properties and batch-to-batch variations are usually not ideal, limiting their biological and biomedical applications 14, 15, 98. Additionally, the difficulties involved in purification and pathogen transmission are also problems to be considered.

#### Synthetic polymers

Compared with natural biomaterials, hydrogels based on synthetic polymers exhibit highly tunable mechanical properties, in addition to the ideal biodegradability, biocompatibility, and biochemical characteristics. Moreover, synthetic polymers, owing to their enhanced controllability and reproducibility, enable the systematic study of cell-matrix interactions 99.

Since Wichterle and Lim first reported a hydrogel for contact lens application in 1960 based on a crosslinked poly-2-hydroxy-ethylmethacrylate (PHEMA) hydrogel by use of ethylene glycol dimethacrylate (EGDMA) 100. Since then, significant progress has been achieved, and a diverse range of synthetic polymers has been used in the field of hydrogel fabrication. In particular, some examples of synthetic polymers that have been studied for the development of injectable hydrogels in cartilage tissue engineering include poly(ethylene glycol) (PEG), poly(vinyl alcohol) (PVA), poly(lactic acid) (PLA) and polydioxanone (PDS), and poly(2-hydroxypropyl methacrylamide) (PHPMA), among others. Copolymers including poly(N-isopropyl acrylamide) (PNIPAAm), P(PEG-co-peptides), and P(PLGA-co-serine) are also routinely employed. Notably, it has been reported that hydrogels based on synthetic polymers have been designed to have similar mechanical properties (compressive modulus) and frictional behavior as articular cartilage. Furthermore, a number of studies have demonstrated that it is easy to encapsulate cells and cytokines into hydrogels formed from synthetic polymers.

PEG is one of the most extensively investigated nondegradable, synthetic, hydrophilic polymers for the development of injectable hydrogels owing to its hydrophilicity, nontoxicity, good tissue compatibility, and availability of reactive end groups for chemical functionalization. Moreover, PEG can be associated with other compounds, such as polycaprolactone (PCL) or poly(lactic-co-glycolic acid) (PLGA), to form block polymers 101.

PVA is a water-soluble polymer, and its solutions can gradually form a gel when stored at room temperature, but with a low mechanical strength 40. Interestingly, once the aqueous solutions of this polymer undergo a freeze-thawing process, a strong and highly elastic gel is formed 40, 102. However, PVA cannot degrade *in vivo* in a relevant time, resulting in limited host integration and an inhibition of tissue ingrowth 38, 103.

PNIPAAm is an inverse temperature-sensitive polymer derived from polyacrylic acid, as well as the most commonly used temperature responsive polymer that shows a sharp phase transition in water at 34.3 °C, close to physiological temperature 39, 40. However, linear PNIPAAm is not stable at physiological temperature, thus requiring the modification of other polymers to improve the stability and mechanical properties 39.

Some disadvantages of these polymers, such as the lack of biological cues for biological applications, especially for proliferation of cells and tissue regeneration, have pushed researchers to find suitable solutions 14, 15. One of the common strategies for this problem is to modify or combine synthetic biomaterials with bioactive polymers. For instance, Yan and colleagues have fabricated a series of injectable poly(L-glutamic acid)/alginate (PLGA/ALG) hydrogels by self-cross-linking hydrazide-modified poly(L-glutamic acid) and aldehyde-modified alginate 39, 104. This injectable PLGA/ALG hydrogel exhibits attractive properties for future application in cartilage tissue engineering. Furthermore, the combination systems of natural and synthetic polymers can almost perfectly make up for the deficiency of the two polymers.

#### Hybrid synthetic/natural polymers

The combination of synthetic and natural polymers has emerged as a promising approach to create injectable hydrogels, combining their inherent superiorities and making up for their shortcomings. Currently, several polymer composites have been reported, including collagen-acrylate, P(HPMA-g-peptide) and HA-g-PNIPAAm 14, 15, 98. These hybrid polymer-based hydrogels, combining the potential chondrogenic tunable characteristics of both synthetic and natural polymers, can be designed to mimic key aspects of the native environment while precisely adjusting the hydrogel's mechanical, chemical and degradation properties 35, 105. For example, Yu et al. fabricated two HA/PEG-based injectable hydrogels, both of which possess good mechanical properties and short gelation times, and the cells encapsulated in the hydrogels exhibit high metabolic viability and proliferation, thus indicating that both hydrogels have great potential in cartilage tissue engineering 39, 106. Additionally, Fu et al. have prepared a novel injectable hydrogel that responds to thermal stimuli and comprises three components (triblock PEG-PCL-PEG copolymer, collagen, and nanohydroxyapatite) 107. This hydrogel composite has a good interconnected porous structure in addition to excellent thermos-sensitivity 107. Moreover, Lin et al. have designed an injectable hydrogel using composite polymers comprising poly(lactic acid-co-glycolic acid)-g-PEG and hydroxyapatite for its promising development in tissue engineering. The addition of hydroxyapatite into the hydrogel enhances the mechanical properties and bioactivity of the hydrogel 39.

### Gelation and degradation of injectable hydrogels

#### Gelation of injectable hydrogels

**Figure 3:**
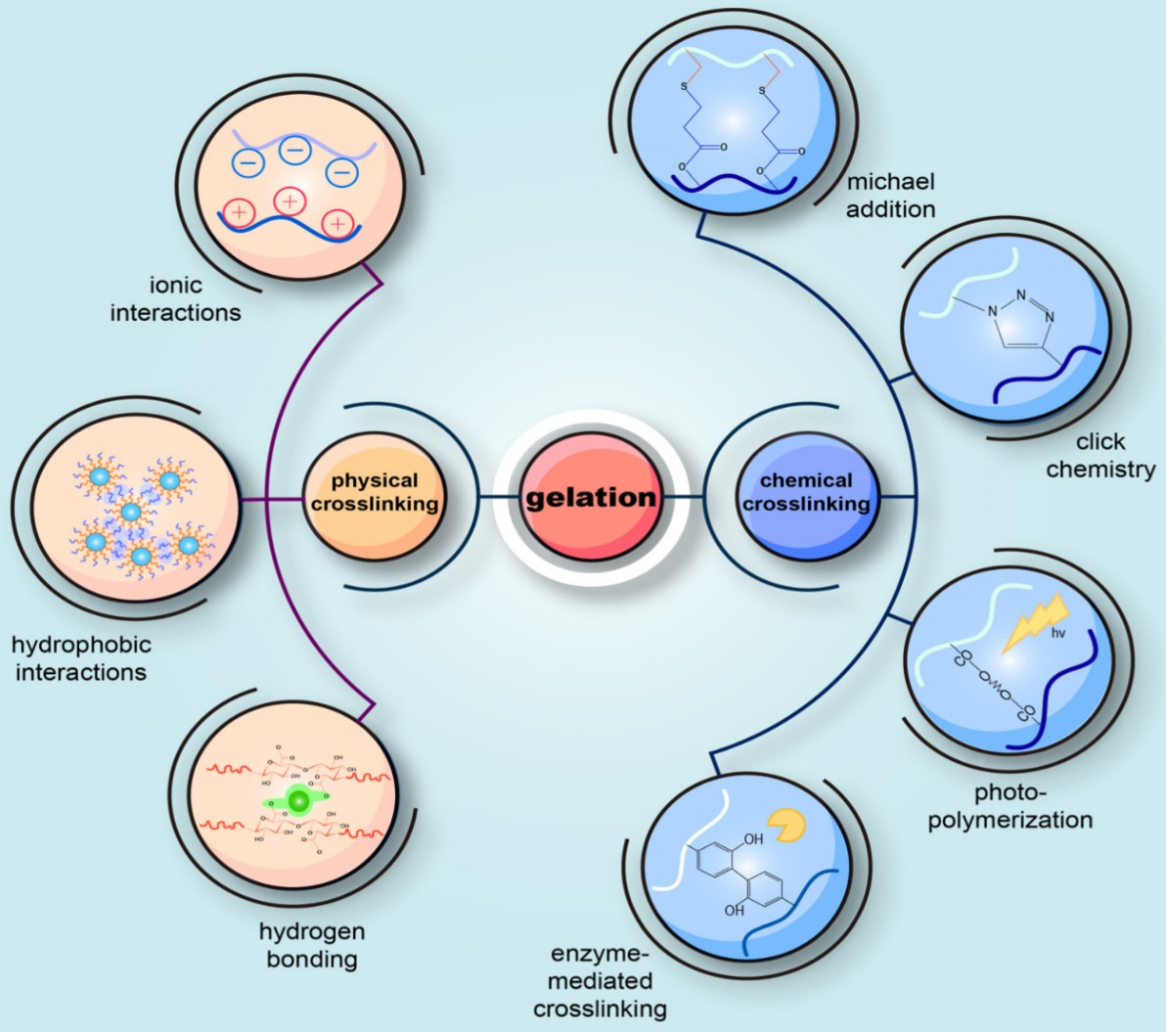
** Schematic diagram of the common methods to prepare injectable hydrogels.** The current gel methods for injectable hydrogels can be broadly classified into two categories: physical crosslinking reactions and chemical crosslinking reactions. One distinction between them lies in the formation of covalent bonds or not. Physical crosslinking is the physical gelation through the emergence of reversible and transient junctions, including molecular entanglements and secondary forces (hydrogen bonding, electrostatic interactions, ionic or hydrophobic interactions). Chemical crosslinking is characterized by the formation of covalent bonds during a variety of chemical processes, including Michael-type addition, click chemistry, enzyme-mediated crosslinking, photo polymerization, the Schiff-base chemistry, and the use of crosslinking agents.

Gelation is an essential step for injectable hydrogels to form a 3D scaffold. The choice of a suitable gel method for the preparation of injectable hydrogels in accordance with the designed structure and the desired application is similarly critical. The current gel methods for injectable hydrogels can be broadly classified into two categories: chemical crosslinking reactions and physical crosslinking reactions (**Figure 3**). One distinction between them lies in the formation of covalent bonds or not.

##### Chemical crosslinking

Chemical crosslinking is characterized by the formation of covalent bonds during a variety of chemical processes, which involve Michael-type addition, click chemistry, disulfide crosslinking, silanization, enzyme-mediated crosslinking, photo polymerization, Schiff-base chemistry and the use of crosslinking agents.

***Michael addition:*** Michael addition, a nucleophilic addition of a carbanion (or nucleophile) to an unsaturated carbonyl residue (e.g., aldehyde or ketone), is commonly used to prepare injectable hydrogels. HA, chitosan, and PEG are usual polymers used to fabricate injectable hydrogels through Michael addition for cartilage repair 108-111. This method is frequently used, owing to its reaction under physiological conditions and its controllable reaction time 110-112. However, in some cases, its slow gelation process limits its clinical application 108.

***Click chemistry:*** Click chemistry refers to a synthetic concept that encompasses a series of reactions including copper-catalyzed azide-alkyne cyclo-addition reactions, Diels-Alder reactions, the thiol-ene reactions, thiol-epoxy, thiol-maleimide couplings, and tetrazine-nor-bornene chemistry. Among them, the first two are the most common routes 113. Due to their rapid polymerization kinetics and low reactivity with cellular components, these reactions are promising for the development of injectable hydrogels 114, 115.

***Enzyme-mediated crosslinking****:* Utilizing enzyme to form covalent bonds and then mediate gelation has drawn increasing attention for its highly biocompatibility, controllable gel time, high site specificity, ability to work at normal physiological conditions, and low cytotoxicity 116. Horseradish peroxidase (HRP) is the most common enzyme to catalyze the reaction and can covalently bind the phenol-conjugated polymers to the ECM proteins of the surrounding native tissue, thus maintaining the structural integrity of the wound tissue 117. It has been reported that several enzyme-mediated crosslinking systems have been applied to prepare injectable hydrogels for cartilage repair, including transglutaminase, tyrosinase, phosphopantetheinyl transferase, lysyl oxidase, plasma amine oxidase, phosphatase, thermolysin, β-lactamase, and peroxidase 118.

***Schiff-base crosslinking****:* The Schiff-base chemistry is a reaction involving an aldehyde (or ketone) and an amine to form an imine (or Schiff base) 38, which has no requirement for external stimuli or additional reagents under physiological conditions. Moreover, it has been incorporated in synthesizing injectable hydrogels for cartilage issue engineering, owing to the mild reaction conditions and high reaction rate. Several polysaccharides such as HA, chitosan and chondroitin sulfate usually apply this method to gelation 38.

***Photo-polymerization****:* Visible or near ultraviolet (UV) light-induced photopolymerization is one of the most extensively investigated gelation processes for developing injectable hydrogels and is a complex process relying on the interaction between visible or UV light and a photo initiator to generate free radicals and polymerization of the radical chain 119. Recently, it has been shown that as the ability to control the timing and location of cross-linking under physiological conditions increases, photopolymerization methods are expected to have a promising application in the field of injectable hydrogels for cartilage tissue engineering 120.

In addition to the methods described above, chemical gelation mechanisms for injectable hydrogels include: crosslinking by formation of disulfide bonds, silanization, and special crosslinking agents, which are not described in detail in this review. In conclusion, the crosslinking chemistry requires the presence of specific functional groups inherently present in the polymer structure or introduced through various chemical modifications 52. Chemical cross-linking can realize gelation* in vivo* through many kinds of chemical reactions in which it is unnecessary to add external induction factors.

##### Physical crosslinking

In the case of physical crosslinking, hydrogels have transient junctions that arise either from polymer chain entanglements or physical interactions such as ionic interactions, hydrogen bonds, hydrophobic interactions or crystal formation 37.

***Crosslinking by ionic interactions:*** Oppositely charged ions exhibit electrostatic interactions, which can lead to the formation of hydrogels *in situ* 52, 121. Hydrogels formed in this way can be divided into two types: ion-embedded hydrogels and polyelectrolyte complexes. The former is formed by the polyelectrolyte bonding with polyvalent ions with opposite charges, while the latter is formed by the interaction of two polyelectrolytes with opposite charges. In particular, the most studied ion-responsive materials for hydrogel fabrication are alginates. Alginates consist of two monomeric units: (1,4)-linked β-D-mannuronate (M) and α-L-guluronate (G) residues 37. In the presence of multivalent cations, such as Ca^2+^, Co^2+^, Sr^2+^, Ba^2+^, Fe^2+^, alginates can realize gelation, which is associated with the binding of cations by G blocks and the formation of a so-called “egg-box” 122. One of the most frequently used ionic agents is calcium chloride (CaCl_2_); however, due to its excellent water solubility, it can cause too rapid and uncontrolled gelation 37, 123. Fortunately, we can retard the gelation progress by adding phosphates and utilizing agents with poor water solubility 122, 123. Furthermore, alginate is a polyanionic electrolyte that can form a polyelectrolyte complex with polycationic electrolytes such as chitosan through electrostatic interactions, which are affected by many factors, such as solution pH and ionic strength 124-126.

***Crosslinking by crystallization:*** Some polymers present a random coil structure in the solution, which can be destroyed and entangled to form a spiral or order structure as the temperature increases or decreases. This is the principle of this physical gelation and it is a reversible process mainly due to the formation of crystallites 127. Natural polymers such as gelatin and polysaccharides can form hydrogels by crystallization. Most natural polymeric materials cool down to form a gel phase, while some aqueous solutions of cellulose derivatives form a gel when heated.

***Crosslinking by hydrogen bonding:*** Intramolecular and intermolecular hydrogen bonding in the aqueous polymer system can also function as a physical crosslinking point. K. Nam et al. obtained two copolymers of methacryloyloxyethylphosphocholine and methacrylic acid and butyl methacrylate. Due to the hydrophobic induction of the butyl group, the carboxyl group forms a carboxyl-carboxyl group and a carboxyl-water-hydrogen bond on the surface to form a physical crosslinking point. These two phospholipid copolymers can automatically form a gel in water.

***Crosslinking by hydrophobic interactions:*** Amphiphilic graft polymers and block copolymers are capable of forming a gel by the association of hydrophobic moieties in the polymer in water. Hydrophobic groups of hydrophobic-modified hydrophobic polymers can form intramolecular intermolecular interactions, and when the concentration is relatively high, an intermolecular association will prevail, forming a polymer network, namely, hydrogels.

***Supramolecular chemistry:*** Supramolecular chemistry is a recently emerging method for developing new injectable hydrogels that has been defined as “chemistry beyond molecules” 38. Although described as supramolecular chemistry, this method is substantially based on the noncovalent binding of molecular motifs through hydrogen bonding, π-π interactions, van der Waals forces, metal chelation and hydrophobic interactions 128. Supramolecular assemblies can produce adjustable, versatile, highly specific and reversible formulations and can generalize some of the biological signaling and structural cues that occur in the body 128. A common example of a supramolecular hydrogel is the association between β-cyclodextrin and another compound such as PEGylated doxorubicin or adamantane 129.

Other physical crosslinking strategies involve fairly special mechanisms, such as stereo-complexation 7, 130. In stereo-complexation, hydrogels are formed by stereocomplexes comprising oligomers of opposite chirality. Specifically, stereocomplex formation occurs, for example, between poly(D-lactic acid) (PDLA) and poly(L-lactic acid) (PLLA), homopolymers of D- and L-lactic acid, respectively 130. Wang and coworkers have designed thermogels fabricated by stereocomplex 4-arm PEG-PLA (scPLA_gel_) and stereocomplex cholesterol-modified 4-arm PEG-PLA (scPLA-Chol_gel_) 131. The resulting scPLA-Chol_gel_ shows lower critical gelation temperature, higher mechanical strength, larger pore size, better chondrocyte adhesion and slower degradation compared to scPLAgel as the benefit of cholesterol modification, which is more appropriate for cartilage regeneration and provides an alternative solution to clinical cartilage repair. An additional form of complexation mechanisms is the formation of inclusion complexes, which is also called guest-host complexation. For example, cyclodextrins have been investigated as building blocks for supramolecular chemistry because they can form complexes with a number of different molecules and polymers through host-guest interaction 132. In this approach, hydrophilic polymers are derivatized with cyclodextrin units, which, upon mixing with a guest molecule-derivatized polymer, result in the formation of a hydrogel structure 130.

In contrast to chemical cross-linking, physical gelation responds to minor changes in temperature, pH, and ionic concentration or strength, which are essential factors for the success of gelation 15, 133. Notably, injectable hydrogels that are sensitive to temperature changes have recently attracted substantial attention for applications in cartilage tissue engineering due to their gelation ability at physiological temperature 39, 131, 134, 135. Hydrophobic interactions and hydrogen bonding play essential roles in the cross-linking mechanism of these hydrogels. Moreover, chitosan is a pH-sensitive polymer that can be dissolved in acidic conditions and further mixed to a weak base to form a physical gel at pH ≈ 6.5 136.

##### Comparison of chemical and physical gelation

There are several criteria necessary for the fabrication of an ideal *in situ*-formed injectable gel applied in biomedical areas, which include: 1) solubility in aqueous solution gelled under physiological conditions (temperature, pH, and ion concentration); 2) no harmful byproducts are released after gelation; and 3) the rate at which gelation occurs is fast enough for clinical efficacy. However, in the presence of additives such as cells and/or bioactive molecules, there is sufficient time for proper mixing and injection 39. Therefore, both physical gelation and chemical gelation need to meet the above criteria. However, both physical and chemical methods have their advantages and disadvantages; therefore, one should choose the most proper method in terms of the design and application of injectable hydrogels.

Physical crosslinking of the polymers is generally considered to have milder conditions than the chemical crosslinking reactions. Minor changes in physical conditions (such as temperature, pH, ion strength, and concentration) promote physical gelation, without chemical reactions, avoiding some biocompatibility issues regarding the use of initiators, monomers or catalysts commonly used for chemical cross-linking 38. However, the low mechanical properties caused by the instability and possible reversibility of these systems exert a negative effect on their application 137. In addition, some strategies, such as those employing a pH-sensitive chitosan hydrogel, are hardly compatible with cell encapsulation 38.

Notably, the injectable hydrogels can be injected via the shear-thinning process if the hydrogels are crosslinked by weak and reversible physical crosslinking, such as coordination bonds and host-guest complexation 138-140. Shear-thinning is a promising technique for the application of injectable hydrogels, where preformed hydrogels can be injected by application of shear stress (during injection) and quickly self-heal after removal of the shear 141. In the traditional method, the initial precursor solution of these hydrogels is injected into the desired area and then crosslinked* in situ* using UV, enzymes, ions, or temperature, while shear-thinning essentially involves injection of preformed solid hydrogels 142. Furthermore, shear-thinning hydrogels provide several advantages over other injectable systems. The shear-thinning technique enables a preformed hydrogel with desired physical properties, and the effect of the local environment on cross-linking is almost negligible, whereas other injectable hydrogels are liquid prior to injection and may be affected by the *in vivo* environment during cross-linking 143. Moreover, the injection of prefabricated hydrogels has little negative impact on the viability and chondrogenesis of the encapsulated cells and subsequent neocartilage development 138. Additionally, in shear-thinning hydrogels, the recovery of elastic modulus after shear (self-healing) may be much faster than the gelation process of other types of hydrogels 144, 145. Furthermore, it has been proved that hydrogels with better shear-thinning properties mediate a more sustained release of small molecular (kartogenin) and proteinaceous (TGF-β1) chondrogenic agents, leading to enhanced chondrogenesis of the encapsulated human BMSCs *in vitro* and* in vivo* 138.

Compared to physical crosslinking, chemical crosslinking generally yields more stable hydrogels with better mechanical properties. The main drawback of chemical crosslinking is the toxicity problems that may be carried by incorporated reactive compounds and/or photoirradiation. Fortunately, recent advances in chemical crosslinking methods have led to the development of systems that undergo gelation under mild reaction conditions.

Advanced fabrication methods require further development, primarily to improve the mechanical properties and physiological stability, to guarantee that gelation occurs at a suitable rate for clinical procedures and to decrease the cytotoxicity and adverse effects of the hydrogels* in vivo*. In short, each approach has its own pros and cons. How to make the right decision about the proper method and improve the current fabrication ways will be explored in future studies.

#### Degradation of injectable hydrogels

Degradation of injectable hydrogels is as important as gelation and plays a critical role in the synthesis of ECM during cartilage tissue growth. Hydrogel degradation may occur through two predominant mechanisms: bulk degradation (e.g., hydrolysis), leading to uniform degradation of the crosslinks, and local degradation (e.g., enzymatic) 35. Natural polymers, especially proteins and proteonucleotides, are usually degraded by enzymatic methods, while synthetic polymers can be designed with crosslinks that degrade by either hydrolysis or enzymes 146. Studies have demonstrated that the rate of hydrogel degradation depends on the degree of crosslinking and the choice of degradable linker 147. Obviously, the more crosslinked, the slower the degradation. However, it is a serious challenge for researchers to balance the degree of crosslinking and the rate of degradation. As a high crosslink density is necessary for joint loads, it is impossible to reduce the degree of crosslinking just for the degradation. A degradable linker that influences the kinetics of degradation and the solute diffusion coefficient is expected to be a solution. Notably, researchers have proposed that by carefully tuning the initial properties and formulation of the hydrogel, it is possible to match degradation with new cartilage tissue growth. Furthermore, to non-invasively monitor hydrogel degradation and efficiently evaluate cartilage restoration *in situ* is still challenging 148.

### Cells and bioactive molecules incorporated in injectable hydrogels

The standard concept of tissue engineering is to incorporate proper cells into a 3D biomaterial scaffold that mimics the native microenvironment of damaged tissues, thus helping regenerate the impaired tissues 35. Injectable hydrogels act as a scaffold that promotes cell-matrix interactions and cell-cell interactions, while bioactive molecules act as a signal that mediates cell migration and adhesion to the scaffold. Therefore, cells and bioactive molecules are important for the injectable hydrogels applied in tissue engineering, both of which play essential roles in cell differentiation and tissue growth.

#### Cell source and cell encapsulation

Incorporation of cells into the injectable hydrogels is critical for regeneration. Cells can either infiltrate into the scaffold, or exogenous cells can be delivered within the scaffold upon implantation 149. The requirements for cells encapsulated into injectable hydrogels applied in cartilage repair are mainly as follows: 1) they are suitable for clinical application, that is, they come from a wide range of sources, are easy to draw from and cause little trauma; 2) they have the ability to form cartilage tissue; and 3) they can achieve the required number of cells after multiple passages and simultaneously maintain the cartilage phenotype 35. Encapsulating cells into injectable hydrogels can be performed by seeding the cells onto prefabricated porous scaffolds, or the cells are encapsulated during scaffold formation. Multiple cell lines have been investigated for cartilage repair, including chondrocytes, mesenchymal stem cells (MSCs), adipose-derived MSCs, and induced pluripotent stem cells (iPSCs) (**Table 2**).

***Chondrocytes:*** The first commonly used cells to repair cartilage damage are autologous chondrocytes, which are amplified *in vitro* and then implanted into damaged cartilage. ACI has been used for decades in the treatment of focal chondral lesions, with good clinical outcomes. Many reports indicate that chondrocytes can proliferate well in hydrogels and express cartilage-related proteins or genes with well-maintained cell morphologies and phenotypes 150-154. Jin and coworkers have developed an injectable hydrogel based on chitosan and found that it could support long-term chondrocyte survival and could retain cell morphology* in vitro* 69. Jin et al. also designed hydrogel based on HA-PEG that exhibited good compatibility with over 95% viable chondrocytes and enhanced production of GAGs and hyaline-specific collagen II for three weeks 52, 108. Moreover, Roberts et al. have proved that a chondrocyte-laden hydrogel consisting of oligo(lactic acid)-b-PEG-b-oligo(lactic acid) can improve the formation of a cartilage matrix consisting of aggrecan and collagen types II/VI 154, 155.

However, obtaining cells from the original cartilage tissue may lead to a secondary cartilage defect in the donor site 154. In addition, the number of chondrocytes available is limited and cannot satisfy the need for tissue engineering and requires long-term* in vitro* culturing (~ 1 month) 156, 157. Furthermore, chondrocytes tend to dedifferentiate into a fibroblast-like phenotype 158. Currently, third-generation ACI, or matrix-induced autologous chondrocyte implantation (MACI) techniques, have incorporated scaffolds to prevent the dedifferentiation of chondrocytes during culture and exhibit positive outcomes in the treatment of focal chondral lesions in the knee 35. However, there are still some problems in donor-site morbidity and decreased effect with increased donor age 154. Allogeneic or xenogenic chondrocytes can also be considered as candidates to repair the cartilage. However, transplantation of allogeneic or allogeneic chondrocytes *in vivo* also has obvious limitations, such as the risk of immune rejection and disease transmission, thus limiting their clinical application 159.

***Stem cells:***Since chondrocytes show insuperable limitations in the treatment of damaged cartilage, a great deal of research has focused on the application of stem cells in recent years. The so-called stem cells refer to a class of pluripotent cells with self-replication ability, which can differentiate into multiple functional cells under certain conditions. Stem cells can be divided into two types according to their source: embryonic stem cells (ESCs) and tissue stem cells. The former, belonging to omnipotent stem cells, can differentiate into almost all cells of the human body. The latter can form more than one type of tissue cell and are pluripotent stem cells. Several stem cell sources have shown the ability to undergo chondrogenesis *in vitro* when seeded in hydrogels, including ESCs, MSCs, and the more recently discovered iPSCs.

**ESCs:** ESCs are highly undifferentiated cells with developmental pluripotency. It has been demonstrated that the combination of these cells with biomimetic hydrogels and growth factors is a synergistic environment for chondrogenesis 160. Hwang et al. have reported the encapsulated ESCs can differentiate into chondrogenic cells and promote the production of neocartilage ECM 160.

Despite promising results, the ethical controversy surrounding their origin is highly scrutinized, which limits their application 161. Furthermore, ESCs are still in the early stage of development and have a strong dependence on the surrounding environment. When the conditions are not suitable, they are prone to malignant transformation and can acquire tumorigenicity, which has been confirmed by mouse experiments. Therefore, there is still a long distance from ESCs that can be cultured *in vitro* to chondrocytes for transplantation applications for the repair of cartilage defects.

**MSCs:** Studies have shown that MSCs derived from adult tissues such as bone marrow, synovium, periosteum, and adipose tissue, as well as umbilical cord and peripheral blood, can differentiate to chondrocytes after culturing in certain conditions 162. MSCs have become the most attractive stem cells in a biomedical area due to their abundant cell sources, low immunogenicity, no ethical concerns and minimal teratoma risk 163, 164. Additionally, MSC-derived exosomes are important in intercellular mitochondria communication and are proved to have therapeutic effect for early osteochondral defect 165. Moreover, agarose, HA, PEG and alginate-based injectable hydrogels encapsulated with MSCs have exhibited the capacity for chondrogenic differentiation in the targeted reconstruction of cartilage 149, 154, 166, 167. Notably, heterogeneity of MSCs influences their application in tissue engineering 168. Bone marrow-derived mesenchymal stem cells (BMSCs) have attracted great interest in the study of articular cartilage tissue engineering 169. Adults' BMSCs can differentiate into connective tissues, including bone, cartilage, muscle, tendon, fat and bone marrow matrix, under certain conditions, thus proving to be the main repair cells after bone and cartilage damage in animals. Simultaneously, BMSCs with strong proliferative ability are easy to isolate from bone marrow and purify *in vitro*, increasing the convenience for autologous transplantation 35. Moreover, it has been proved that CD146^+^ subpopulation from adipose-derived mesenchymal stem cells (ADSCs) play essential roles in promoting cartilage repair 168. And composite scaffold combined with synovium-derived mesenchymal stem cell (SMSC) could greatly strengthen chondroprotection 170. Therefore, MSCs have been considered as the most promising cells in cartilage tissue engineering in recent years, methods to improve the chondrogenesis of which are feasible and could potentially be achieved using injectable hydrogels, which would provide them with strong chondrogenic cues and prevent their differentiation toward unwanted tissues.

**iPSCs:** The recently discovered iPSCs are obtained from the reprogramming of adult cells, staying at an early state of differentiation that resembles ESCs 171. It has been demonstrated in a rabbit model that human iPSCs can maintain their pluripotency in a poly-lactic-based scaffold and enhance cartilage repair of an osteochondral defect in a 6-week period 172. With the advancement of cellular reprogramming techniques, it is now possible to generate iPSCs using an integration-free approach, which is safer and more amenable from a regulatory perspective for their eventual clinical use 35, 173. Although iPSCs show positive outcomes in application, further studies are needed to better assess the long-term benefits of using human iPSCs for articular cartilage tissue engineering.

Over the last decade, various types of cell-loaded injectable hydrogel systems have been investigated for cartilage regeneration 30, 122. However, there are some problems regarding encapsulated cells that limit their development. We are not certain about the exact pathways of chondrogenic differentiation, especially during the progress of cartilage repair. Therefore, the first challenge is the dedifferentiation of the generated chondrocyte phenotype, resulting in easy degeneration and fibrosis of tissue-engineered cartilage in the later stage, with little collagen and proteoglycan remaining. Furthermore, the type of cells involved in the design of injectable hydrogels largely determines the characteristics of the regenerated tissue 52. For instance, autologous chondrocyte therapy generates better hyaline-like cartilage compared to microfracture-based mesenchymal cell therapy 174. Based on the expected tissues' characteristics, choosing proper cell types to encapsulate in hydrogels is another challenge. Additionally, the success of cell therapy requires the effective localization and retention of transplanted cells at the site of injury 144, 175. Injectable hydrogels augment the uniformity in distribution of cells throughout the scaffolds for advantages in *in situ* formation. However, most cell-based therapies require a two-step approach: first, harvesting the cells; and second, expanding the cell population 35. From the perspective of FDA regulations, this manipulation of tissues and cells is considered beyond minimally manipulated and requires greater regular oversight, which may delay its translation to the clinic 176.

#### Bioactive molecules

In the studies of the mechanism of cartilage metabolism, bioactive molecules, including cartilage matrix macromolecules and related regulatory factors, play essential roles after the damage of cartilage 177. Under physiological conditions, the metabolism of cartilage tissue is in a dynamic equilibrium, and the biomacromolecules involved in maintaining this dynamic balance include metalloproteinases (MMPs), interleukin-1(IL-1), and transforming growth factor-β (TGF-β). However, the expression and relationship of these biomacromolecules during the repair process have remained mysterious for researchers.

Studies have confirmed that many cytokines or growth factors play important roles in the proliferation and metabolism of chondrocytes and the differentiation of stem cells into chondrocytes. The growth factors that are currently receiving large amounts of attention in cartilage tissue engineering mainly include TGF-β, bone morphogenetic protein (BMP) and insulin-like growth factor (IGF). These growth factors have been explored alone or in combination for cartilage regeneration.

***TGF-β:*** TGF-β is a secreted multifunctional protein that can regulate cell proliferation, differentiation, and extracellular matrix metabolism, thus playing an important role in the synthesis and metabolism of chondrocytes 164. Chondrocytes in a biodegradable injection hydrogel encapsulated with TGF-β1 showed proliferation over 21 days with minimized dedifferentiation 123. In addition, it has been found that TGF-β can promote chondrogenic differentiation of BMSCs during chondrogenic induction 64, 65. On culturing periosteum in polysaccharide gels such as agarose and alginate with TGF-β1, the proliferation and differentiation of chondrogenic precursor cells in the cambium layer promoted chondrogenesis 178.

***BMP:*** BMP describes a group of highly conserved functional proteins with similar structures that can stimulate the synthesis of DNA and the replication of cells, thus promoting the targeted differentiation of mesenchymal cells into osteoblasts. BMP is also a major factor inducing bone and cartilage formation *in vivo*, is expressed in limb growth, endochondral ossification, early fracture and cartilage repair, and plays important roles in embryonic development and regeneration repair of bone 66.

***IGF:*** IGF is a multifunctional cell proliferation regulator with the ability to promote cell differentiation and proliferation, similar to insulin 68. IGF-1, also known as somatomedin C, primarily acts in an anabolic fashion to increase the synthesis of proteoglycan and collagen II 179. Growth factors loaded in the scaffold facilitate localization and continuous delivery and mediate important cellular processes, including progenitor cell differentiation, proliferation, and ECM synthesis 52, 179.

Due to the different biological effects of various growth factors, there is a tendency to use a variety of growth factors in combination at present. For instance, co-encapsulation of TGF-β3 and basic fibroblast growth factor (bFGF) in an injectable thermos-responsive hydrogel promoted chondrogenic differentiation of rabbit MSCs by mediating molecular and cellular processes that resemble *in vivo* chondrogenesis 180. Injectable gellan gum combined with TGF-β1 and BMP-2 preconditioned chondrogenic ASC-enhanced collagen II and aggrecan expression while down-regulating collagen I, thereby emerging as a promising construct for cartilage repair 52, 181.

However, there also remain some problems, particularly involving growth factors. Growth factors in the body, with their short half-lives, are soon cleared from the local body and cannot mediate the ideal effect. Furthermore, different concentrations of growth factors probably lead to opposite effects. Moreover, the concentration of the growth factors is hard to control, and excessive concentrations inhibit the formation of cartilage and reduce the effect 182, 183. Finally, growth factors exhibit potential immunogenicity, and repeated injection can possibly induce synovitis and synovial hyperplasia 184, 185.

## A novel injectable hydrogel: Hybrid hydrogel combined with Nanoparticles

In bone or cartilage tissue engineering, the bearing capacity of the material is an essential feature. However, the stiffness of hydrogel scaffold is 2 orders of magnitude lower than that of natural cartilage, which is the major defect in the development of cartilage regeneration 186. Although the methods of changing polymer concentration, copolymerization or mixing with different polymers can effectively control the degradability, swelling behavior or gelation temperature of hydrogels, there are some limitations in improving the mechanical properties or cellular reactions of hydrogels 187, 188. Currently, researchers report that these obstacles can be alleviated through nanomaterials added to hydrogel scaffolds 189. Therefore, nanocomposite hydrogels have attracted much attention in recent years.

Hybrid hydrogels integrated with nanoscale composites are defined as hydrated polymeric networks that are either physically or chemically cross-linked with nanoparticles or other nanostructures 190. Different types of nanoparticles, including carbon-based nanomaterials (such as carbon nano-tubes, graphene, and nanodiamonds), inorganic/ceramic nanoparticles (such as hydroxyapatite, silica, silicates, and calcium phosphate), polymeric nanoparticles and metal/metal oxide nanoparticles (such as gold, silver, and iron oxide), can be incorporated into the polymeric network to form nanocomposite hydrogels 154, 190.

Nanoscale composites with large surface area-to-volume ratios can not only improve the surface reactivity but also provide enhanced mechanical properties 154. For instance, Boyer and coworkers have developed a hybrid interpenetrating network mixed with a nanoreinforcing clay (laponites) and silated hydroxylpropyl-methyl cellulose, which increased the hydrogel mechanical properties without compromising its oxygen diffusion capability, cytocompatibility, the self-organization of chondrogenic cells and generation of ECM components 191. It has been demonstrated that both ceramic and polymer additives can provide significant mechanical reinforcement of hydrogel.

Another function of the added nanocomposites is biological activity and biomimetic function; to mimic the ECM of cartilage, several nanomaterials have been encapsulated into the network of injectable hydrogels. For example, Zhang et al. designed a hybrid hydrogel (MagGel) comprising type II collagen, HA, PEG, and magnetic nanoparticles for cartilage regeneration 192. Notably, the MagGel showed similar microstructure and chemistry to hyaline cartilage and was cytocompatible with BMSCs *in vitro* 192.

Moreover, nanoparticles are desirable formulations for controlled drug release due to their high surface area-to-volume ratios, small dimensions, high drug-encapsulating efficiencies and the capacity to quickly respond to surrounding environmental stimuli, such as temperature, pH, magnetic fields or ultrasound 193-195. Shi et al. have reported an acrylated HA injectable hydrogel with kartogenin (KGN) encapsulated into biodegradable PLGA nanoparticles through an emulsion-based formulation method, which exhibits a sustained release of KGN and facilitates the filling of the defects and generation of hyaline cartilage 196. Additionally, Spiller et al. have designed a hybrid scaffold system comprising PVA and PLGA loaded with IGF-1, resulting in the release of IGF-1 in a linear and sustained manner for at least 45 days 197. In addition, since nanoparticles can easily penetrate into the focal tissue via narrow or small capillaries or the epithelial lining, the efficacy of loaded therapeutic agents and bioactive agents can be enhanced 198-200.

Collectively, nanocomposite hydrogels combine the advantages of injectable hydrogels and nanofillers, which can provide mechanical reinforcement as well as enhancement of the biological activity of hydrogels by ECM mimicry and the possibility of delivering growth factors and drugs.

Despite the promising results (even with a 6-fold increase of storage modulus), the properties of the hydrogel-matrix are still notable, which in most cases does not allow achievement of mechanical properties comparable to those of native cartilage 37. Additionally, future research should focus on functional tests of these hydrogels *in vivo* to characterize the influence of nanofillers on the whole organism, including the risk of side effects.

## Advances of injectable hydrogels applied in cartilage repair

Recently, various injectable hydrogels with good moldability and 3D structures have been widely investigated for use in cartilage tissue engineering. For example, Park et al. successfully fabricated an injectable chitosan-HA hydrogel with encapsulated chondrocytes. The hydrogel showed excellent proliferation and increased deposition of cartilaginous ECM; considering these results, this hydrogel has great potential for cartilage tissue repair 120. Kontturi et al. developed an injectable, *in situ-*forming type II collagen/HA hydrogel for cartilage tissue engineering that has been shown to maintain chondrocyte viability and characteristics and may be a potential injectable scaffold for cartilage tissue engineering 63. More examples are provided in **Table 2.**

Moreover, numerous preclinical studies (including large and small animal studies) have evaluated the effects of injectable hydrogels on cell repair after complexation with cells. These preclinical animal models include rabbit, canine, mini-pig, ovine, caprine, and equine, while cell types include autologous bone marrow mesenchymal stem cells and allogeneic umbilical cord blood mesenchymal stem cells 35. Although there are various distinctions in experimental manipulations (such as cell collection, processing, and delivery), these studies have shown that cartilage regeneration is improved over 3 months to 2 years with encouraging results 201-205.

Though there are numerous preclinical studies on the development of tissue engineered medical products, clinical trials are very limited due to regulatory issues, differences in patients' healing responses, large-scale fabrication and skilled expertise for handling in production as well as implantation constraints 52. Most of the literature includes case reports or case series. In the case-control treatment, researchers have found that the combination of microfracture and hydrogel has a better effect than the treatment of cartilage injury with microfracture alone (**Table 3**) 206, 207.

Overall, both animal and clinical studies have shown greater cartilage regeneration in the hydrogel treatment of cartilage defects than in microfractures alone 35. Injectable hydrogels are a promising therapeutic tool to deliver cells *in vivo* for the treatment of cartilage damage, which is certified by multiple preclinical animal models. Further studies, including blinded randomized control trials, will determine the true clinical effectiveness of hydrogels in cartilage repair.

## Conclusions and Perspectives

There is no doubt that injectable hydrogels are potential tools for cartilage tissue engineering, not only for their biomimetic properties and high moisture content but also for their minimal invasive properties and ability to match irregular defects. In spite of the relatively successful preclinical results and advanced synthesizing methods of several engineered tissues, various significant challenges remain to be addressed in fabricating injectable hydrogels to optimally achieve cartilage regeneration.

The major challenge lies in the perfect design of bioactive scaffolds with excellent biocompatibility, biodegradability, stability, and favorable mechanical properties 208. Furthermore, pore size, interconnections, and porosity of scaffolds in cartilage tissue engineering greatly influence nutrients, metabolites and oxygen diffusion, as well as neovascularization, cellular infiltration/migration, and sufficient space for the tissue in-growth 209, 210. In addition, injectable hydrogels are often hindered by absence of macroporosity and the lack of complex microvasculature, which leads to considerable losses of both viability and function of the seeded cells due to the resulting deficiency in transportation of nutrients and signaling molecules. Notably, although articular injection therapy has been widely applied in osteoarthritis and joint injury, the intra-articular injection has a short duration of action and requires multiple injections, which reduces the patient's compliance. Therefore, how to maintain the medicines effectiveness and reduce patient pain should also be considered in the clinical application of injectable hydrogels. To address these challenges, the development of bioactive biomaterials and advanced preparation methods are required.

The mechanical resilience and rapid recovery abilities of hydrogel implants are critical in load-bearing tissues, such as articular cartilage, which are routinely subjected to cyclic loadings of high magnitude and frequency 211, 212. Fortunately, it has been proven that the energy dissipation by the hydrogels can protect the loaded cells. For example, dynamic single-chain nanogels as building blocks provide effective energy dissipation to bulk hydrogels, thereby protecting the encapsulated stem cells from deleterious mechanical shocks in a 3D matrix 211. Additionally, PEG-adamantane supramolecular hydrogels exhibit substantial deformability and excellent capacity to dissipate massive amounts of loading energy and have a rapid, full recovery during excessive, ultrafast, and nonresting cyclic compression 212.

## Figures and Tables

**Table 1:** Mechanical properties of articular cartilage tissue

Mechanical property	Articular cartilage	Reference
Aggregate modulus (MPa)	0.1-2.0	213-216
Compressive Young's modulus (MPa)	0.24-0.85	37
Tensile Young's modulus (MPa) - constant-strain rate	5-25	213, 217
Tensile equilibrium modulus ( MPa)	5-12	218
Equilibrium shear modulus ( MPa)	0.05-0.4	218, 219
Complex shear modulus ( MPa)	0.2-2.5	218, 220, 221
Equilibrium Relaxation Modulus ( MPa)	6.5-45	37
Compressive Strength (MPa)	14-59	213, 222
Tensile strength (MPa)	0.8-25	223
Ultimate Tensile Stress (MPa)	15-35	37
Shear loss angle (°)	10-15	218, 221, 224
Hydraulic permeability (m4/Ns)	10-16-10-15	214, 222
Elongation at Break	80%	37
Poisson's ratio	0.06-0.3	213, 224, 225

**Table 2:** Examples of injectable hydrogels for cartilage tissue engineering

Gelation	Mechanism	Polymer	Cells	References
Physical crosslinking	Ionic	alginate-graft-hyaluronate	Chondrocytes (mouse)	226
	pH-sensitive	poly(γ-glutamic acid)-PEG	cartilage chondrocytes (bovine)	227
	Thermo-sensitive	Pluronics®; methylcellulose; PNIPAAm-gelatin	Chondrocytes (goat); articular chondrocytes (rabbit); chondrocytes (rabbit)	228-230
Chemical crosslinking	Michael addition	thiolated hyaluronic acid and PEG vinylsulfone; dextran-PEG	Chondrocytes; chondrocytes or embryonic stem cells	108, 112
	Click chemistry	dextran-based hydrogels; HA/PEG hydrogel	Chondrocytes (rabbit); Chondrocytes (mouse)	106, 142
	Enzyme-catalyzed	glycopolypeptides	Chondrocytes	231
	Schiff-base reaction	N-succinyl-chitosan (S-CS) and aldehyde hyaluronic acid (A-HA); poly(ethylene oxide-co-glycidol)-CHO and glycol chitosan (GC)	articular chondrocytes (bovine); chondrocyte (mouse)	71, 232
	Photo-polymerization	methacrylated hyaluronan, methacrylated gelatin	MSCs (humam); hBMSCs	165, 233, 234

**HA**, Hyaluronic acid; **ACM**, acellular cartilage matrix; **BMHP**, bone marrow homing peptide; **SAP**, self-assembling peptide**; PEG**, poly (ethylene glycol); **PNIPAAm**, poly(N-isopropyl acrylamide).

**Table 3:** Applications of some injectable hydrogels for cartilage tissue engineering

Process	Polymer	Cell types	Model	Study	Year	Reference
Small animal study	HA	MSCs	Minipig	Ha et al.	2015	235
	Chitosan	Chondrocytes	Rabbit	Zhao et al.	2015	205
	ACM-BMHP-SAP	MSCs	Rabbit	Lu et al.	2018	236
Large animal study	Fibrin	ES-like cells	Ovine	Pilichi et al.	2014	204
	Fibrin	BMSCs	Equine	Goodrich et al.	2016	201
	Alginate	Chondrocytes or periosteal cells	Sheep	Schagemann et al.	2009	237
	Fibrin	Autologous chondrocytes	Goat	Lind et al.	2008	238
	Aragonite-HA	Not applicable	Goat	Kon et al.	2015	202
	Collagen	MSCs	Monkey	Araki et al.	2015	239
Clinical study	PEGDA-HA	Not applicable	Human	Flisseeff et al.	2013	206
	ChonDux	Not applicable	Human	/	/	38
	GelRine	Not applicable	Human	/	/	38
Commercial	BST-CarGel (Chitosan Scaffold)	Not applicable	Human	Restrepo et al.	2015	207

**BMSCs**, Bone marrow-derived stromal cells; **HA**, Hyaluronic acid; **MSCs**, mesenchymal stem cells.
